# Follicular fluid and serum biochemical and hormonal profiles of normal and cystic dromedary camel breeds

**DOI:** 10.14202/vetworld.2022.2511-2516

**Published:** 2022-11-08

**Authors:** Amal Bekkouche, Kamel Miroud, Nora Mimoune, Brahim Benamor, Rachid Kaidi, Mohammed Hocine Benaissa

**Affiliations:** 1 Department of Veterinary Sciences, Chadli Bendjedid University, Laboratory of epidemio-surveillance, health, productions and reproduction, experimentation and cellular therapy of domestic and wild animals. BP, 73, Eltarf 36000 Algeria; 2 Clinical Department, Animal Health and Production Laboratory, National High School of Veterinary Medicine, Algiers, 16000, Algeria; 3 Veterinary Practitioner, El-Bayadha, El-Oued 39000, Algeria; 4 Clinical Department, Institute of Veterinary Sciences, LBRA, University of Blida 1, PB 270, Soumaa, Blida, 09000, Algeria; 5 Scientific and Technical Research Centre for Arid Areas (CRSTRA), Biophysical Station, PB 30240, Nezla, Touggourt

**Keywords:** camel breeding, follicular fluid, ovarian cyst, serum

## Abstract

**Background and Aim:**

Ovarian cysts (OC) in female dromedary camels have been described as problematic because they can cause infertility. This study aimed to compare the hormone concentrations and biochemical contents present in serum and follicular fluid of normal and cystic she-dromedaries of the two most common Algerian camel breeds (Sahraoui and Targui) to gain a better understanding of biological differences that may yield insights into preventing or treating this ovarian abnormality.

**Materials and Methods:**

At an abattoir in southeastern Algeria, 100 pairs of the same females’ ovaries and blood samples were taken immediately after the slaughter of clinically healthy, non-pregnant females (8–15 years old) over two consecutive breeding seasons (November 2017–April 2018 and November 2018–April 2019). The concentrations of glucose, cholesterol, protein, urea, creatinine, triglyceride, gamma-glutamyl transferase, alanine aminotransferase, and aspartate aminotransferase were determined using commercial diagnostic kits and standard analytical procedures. Electrochemiluminescence immunoassay was used to measure progesterone (P4) and insulin concentrations.

**Results:**

The concentrations of glucose, insulin, cholesterol, and P4 in sera and follicular fluid (regardless of ovarian follicle diameter) were different (p < 0.001), but there was no significant difference in the other parameters studied. Glucose, insulin, cholesterol, urea, and P4 levels in blood serum differed significantly from pre-ovulatory follicles. None of the biochemical and hormonal components measured differed significantly between the pre-ovulatory and cystic fluids of the she-dromedaries studied. The breed did not affect the biochemical and hormonal composition of she-dromedary cystic and follicular fluids.

**Conclusion:**

Ovarian cysts appear to form in a metabolic milieu distinct from follicular fluid and blood serum, with no influence from camel breeds. It is suggested that further research on the blood-follicle barrier be conducted to gain a better understanding of the OC development process in she-dromedaries.

## Introduction

Camel (*Camelus dromedarius*) is uniquely adapted to provide a vital food source and financial stability to impoverished rural communities in arid and semi-arid regions. Camel breeding is economically and socially important in many African and Asian countries [1, 2], including Algeria, where approximately 416,500 dromedaries [3] are concentrated primarily in the arid regions of South Algeria. Despite their importance, camels often face the problem of low reproductive efficiency. She-dromedaries reach puberty at the age of 3, but typically do not reach full sexual maturity until the age of 4 or 5 [1]. The camel breeding season lasts <½ a year and coincides with the rainy season from December to May [4, 5]. Ovarian follicular maturation lasts 6 days, and there is no luteal phase during the normal ovulatory cycle [1]. When ovulation occurs due to the absence of the mating stimulus [1], the dominant follicle reaches a maximum diameter of approximately 4.2 ± 0.2 cm during estrus before regression [6]. During folliculogenesis, the ovarian follicle grows significantly, and the oocyte matures in the follicular fluid, a complex biochemical milieu [7]. Follicular fluid is a mixture of serum exudate and locally produced substances related to the metabolic activity of follicular cells [8]. It provides a critically important microenvironment for normal oocyte maturation and growth [9]. During ovarian follicular development, the metabolic activity and “barrier” properties of the follicular wall differ significantly [8].

Disturbances in hormonal and metabolic balances and various oxidative stresses can lead to the formation and persistence of ovarian follicles, resulting in infertility problems [10]. An ovarian follicular cyst is an abnormally large ovarian follicle on one or both ovaries, representing a physiologic variant of normal follicular dynamics [1] that many consider problematic [11, 12]. Camels have ovarian follicular and luteal cysts, but follicular cysts are more common [10]. Follicular cysts typically contain a yellowish fluid but may contain blood; they are typically 3–5 cm in diameter but can be larger. Luteal cysts appear as a dark red mass packed with a pigmented, semi-coagulated discharge on the ovary’s surface [6].

Ovarian cysts (OC) are caused by metabolic diseases and hormonal imbalances [13]. Because little is known about the etiology and pathogenesis of OC in camels, investigating hormonal and metabolic indicators related to OC are beneficial [14]. Monitoring the ovarian follicular fluid composition and serum profiles in normal and cystic she-dromedaries of various camel breeds could lead to a better understanding of the underlying biological mechanism of OC development.

This study aimed to compare the hormone concentrations and biochemical contents found in serum and follicular fluid of normal and cystic she-dromedaries from the two most common Algerian camel breeds (Sahraoui and Targui).

## Materials and Methods

### Ethical approval

No ethical approval was required for this work because ovaries and blood samples were recovered at the time of postmortem from she-dromedaries at an abattoir. All the animal studies were conducted with the utmost regard for animal welfare, and all animal rights issues were appropriately observed.

### Study period and location

This study was conducted from November 2017 to April 2019 at the main abattoir of Oued Souf (in South east Algeria), The samples were processed at the laboratory of the Scientific and Technical Research Centre for Arid Areas (Touggourt) and the National High School of Veterinary Medicine (Algiers).

### Animals and sample collection

The study was conducted on animals brought for slaughter at Oued Souf’s primary abattoir in Southeast Algeria. A postmortem macroscopic examination after slaughter revealed no anomalies in the reproductive organs. Hence, the animals were declared clinically healthy from a reproductive perspective.

One hundred pairs of ovaries were obtained from non-pregnant she-dromedaries aged 8–15 from two different breeds (Sahraoui, n = 80; Targui, n = 20). Blood samples (10 mL each animal; n = 100 animals) were drawn into test tubes containing ethylenediaminetetraacetic acid as an anticoagulant. Blood samples and ovaries were stored in a cooler at 4°C and transported to the laboratory within an hour of collection, where the ovarian follicles were measured and classified as normal or cystic based on their diameter, normal (≤2.0 cm) and cystic (>2.0 cm) [6]. Ovarian follicles were aspirated using a disposable sterile needle and syringe. Blood samples were centrifuged for 5 min at 2.250 g, and serum and ovarian fluids were kept at −20°C until analysis.

### Biochemical and hormonal analyses

Biochemical analyses were performed on blood samples and ovarian fluids (from follicles and cysts) for glucose, urea, cholesterol, protein, aspartate aminotransferase (AST), triglycerides, creatinine, alanine aminotransferase (ALT), and gamma-glutamyl transferase (GGT). Analyses were performed using spectrophotometric methods on a clinical chemistry auto-analyzer (Architect Plus ci4100, Abbott Rapid Diagnostics GmbH, Köln, Germany). The intra-assay and inter-assay coefficients of variance were <5% in all analyses. The concentrations of biochemical contents were calculated by dividing the test sample’s absorbance by the standard’s absorbance multiplied by the standard concentration. Electrochemiluminescence (Cobas e411 analyzer, Roche Diagnostics GmbH, Mannheim, Germany) was used to calculate insulin and progesterone (P4) concentrations. All measurements were taken following the manufacturer’s instructions.

### Statistical analysis

For statistical data analysis, the XLSTAT software version 2016.02.28451 (Addinsoft, Paris, France) was used. The Chi-square test was used to examine the effect of breed on the incidence of cystic ovaries. Tukey’s test, REGWQ *post hoc* test, or Dunnett’s test were employed for multiple comparisons (depending on the parameter). Pearson’s correlation analysis was used to examine the relationship between ovarian fluid and serum in normal and cystic females and the equilibrium between those two biological liquids in normal and cystic conditions. Data are presented as mean ± standard error of the mean, with differences considered significant at p < 0.05.

## Results

The prevalence of cystic ovaries in this study’s animal population was 33%, and it was not affected (p > 0.05; χ^2^) by breed (27/80 for Sahraoui and 6/20 for Targui) or female age. Table-1 shows the mean biochemical and hormonal values for serum and follicular fluids for all 100 she-camels, considering the breed, age, and ovarian status (normal follicle vs. cyst). The concentrations of glucose, insulin, cholesterol, and P4 differed (p < 0.05) between blood serum and ovarian fluids but not the other seven parameters tested (p > 0.05). As expected, cystic ovarian follicles had a larger diameter (p < 0.001) than normal ovarian follicles (4.66 ± 3.58 cm vs. 1.20 ± 0.64 cm). However, no differences (p > 0.05) were found between normal and cystic she-dromedaries (Table-2). Glucose, insulin, cholesterol, urea, and P4 concentrations in pre-ovulatory follicles and cystic fluid differed significantly from those in blood serum (Tables-1 and 2). Table-3 compares cystic and pre-ovulatory fluids’ biochemical and hormonal profiles of two she-dromedaries breeds (Sahraoui and Targui). Pearson’s correlation analysis revealed a non-significant correlation (p > 0.05) between the parameters in the cystic and pre-ovulatory fluids of the two she-dromedaries breeds.

**Table-1:** Comparison of serum and follicular biochemical and hormonal profile of normal she-dromedaries (mean ± SEM).

Parameters	Blood serum (n = 100)	Follicular fluid (n = 100)	p-value	r
Glucose (mmol/L)	4.82 ± 0.12	1.99 ± 1.99	<0.0001*	0.651
Insulin (µU/mL)	5.33 ± 0.14	0.85 ± 0.064	<0.0001	0.794
Total protein (g/L)	70.07 ± 0.61	62.49 ± 0.79	>0.05	0.225
Triglycerides (mmol/L)	0.56 ± 0.028	0.44 ± 0.014	>0.05	0.065
Total cholesterol (mmol/L)	2.43 ± 0.071	0.17 ± 0.011	<0.0001*	0.832
AST (U/L)	92.57 ± 2.80	107.07 ± 1.54	>0.05	0.094
ALT (U/L)	25.90 ± 1.16	26.84 ± 0.93	>0.05	0.002
GGT (U/L)	31.41 ± 1.25	28.25 ± 1.07	>0.05	0.018
Urea (mmol/L)	3.61 ± 0.11	1.59 ± 0.09	>0.05	0.490
Creatinine (µmol/L)	110.57 ± 2.57	82.02 ± 2.22	>0.05	0.263
P4 (ng/mL)	0.87 ± 0.06	82.25 ± 3.10	<0.0001*	0.777

SEM = Standard error of the mean, r = Correlation coefficient, P value = Pearson’s Chi-square,

* Values with an asterisk within the same raw are statistically significant (p < 0.05), AST = aspartate aminotransferase, ALT = alanine aminotransferase, GGT = gamma-glutamyl transferase, P4 = progesterone.

**Table-2:** Comparison of biochemical and hormonal values of ovarian fluids from normal and cystic she-dromedaries (mean ± SEM).

Parameters	Normal dromedaries (n = 67)	Cystic dromedaries (n = 33)	p-value	r
Follicle diameter (cm)	1.20 ± 0.64	4.66 ± 3.58	0.000*	0.381
Glucose (mmol/L)	2.05 ± 0.72	1.84 ± 0.77	>0.05	0.018
Insulin (µU/mL)	0.97 ± 0.67	0.73 ± 0.56	>0.05	0.042
Total protein (g/L)	62.83 ± 7.72	62.78 ± 8.46	>0.05	0.003
Triglycerides (mmol/L)	0.45 ± 0.13	0.44 ± 0.17	>0.05	0.004
Total cholesterol (mmol/L)	0.18 ± 0.11	0.16 ± 0.10	>0.05	0.005
AST (U/L)	104.97 ± 14.74	106.92 ± 16.9	>0.05	0.020
ALT (U/L)	28.56 ± 9.89	26.40 ± 8.11	>0.05	0.034
GGT (U/L)	28.00 ± 9.55	30.79 ± 12.66	>0.05	0.022
Urea (mmol/L)	1.63 ± 0.93	1.44 ± 0.86	>0.05	0.011
Creatinine (mmol/L)	84.66 ± 21.60	83.17 ± 23.69	>0.05	0.018
P4 (ng/mL)	80.98 ± 29.49	84.66 ± 34.22	>0.05	0.003
Age of animals	11.48 ± 3.65	11.85 ± 4.26	>0.05	0.002

SEM = Standard error of the mean, r = Correlation coefficient, P value = Pearson’s Chi-square,

* Values with an asterisk within the same raw are statistically significant (p < 0.05), AST = aspartate aminotransferase, ALT = alanine aminotransferase, GGT = gamma-glutamyl transferase, P4 = progesterone.

**Table-3:** Comparison of biochemical and hormonal profile in cystic and pre-ovulatory fluids of Sahraoui and Tergui dromedary camel breeds (mean ± SEM).

Parameters	Cystic fluid		Normal follicular fluid	
	
	Breed Sahraoui (n = 27)	Breed Tergui (n = 6)	Breed Sahraoui (n = 53)	Breed Tergui (n = 14)
Follicle diameter (cm)	5.03 ± 3.93^a^	3.72 ± 0.44^a^	1.33 ± 0.64^a^	1.30 ± 0.66^a^
Glucose (mmol/L)	1.83 ± 0.77^a^	1.99 ± 0.85^a^	2.10 ± 0.73^a^	1.93 ± 0.70^a^
Insulin (µU/mL)	0.64 ± 0.48^a^	0.86 ± 0.87^a^	0.90 ± 0.64^a^	1.03 ± 0.79^a^
Total proteins (g/L)	61.39 ± 8.12^a^	67.16 ± 9.06^a^	62.72 ± 7.52^a^	61.73 ± 8.72^a^
Triglycerides (mmol/L)	0.44 ± 0.16^a^	0.42 ± 0.21^a^	0.43 ± 0.13^a^	0.48 ± 0.14^a^
Total cholesterol (mmol/L)	0.15 ± 0.10^a^	0.17 ± 0.10^a^	0.177 ± 0.11^a^	0.17 ± 0.10^a^
AST (U/L)	107.77 ± 17.46^a^	111.58 ± 15.24^a^	108.21 ± 13.74^b^	99.49 ± 16.83^a^
ALT (U/L)	23.92 ± 6.93^a^	31.66 ± 10.63^b^	27.28 ± 10.38^a^	28.71 ± 8.00^a^
GGT (U/L)	29.685 ± 12.12^a^	31.833 ± 16.06^a^	26.87 ± 8.64^a^	29.16 ± 12.66^a^
Urea (mmol/L)	1.481 ± 0.92^a^	1.415 ± 0.56^a^	1.67 ± 0.94^a^	1.58 ± 0.93^a^
Creatinine (µmol/L)	80.986 ± 25.49^a^	80.500 ± 14.61^a^	80.37 ± 17.85^a^	90.91 ± 31.22^a^
P4 (ng/mL)	84.367 ± 33.12^a^	86.333 ± 42.27^a^	81.27 ± 27.22^a^	80.14 ± 38.09^a^

SEM = Standard error of the mean, ^a,b,c^Values within a row with different superscripts differ significantly at p < 0.05,

AST = aspartate aminotransferase, ALT = alanine aminotransferase, GGT = gamma-glutamyl transferase, P4 = progesterone

## Discussion

To the best of our knowledge, this is the first study to investigate the biochemical and hormonal changes in the follicular and cystic fluids of she-dromedaries and the possible effect of breed on OC development. Follicular fluid biochemical metabolites are required for oocyte development and fertilization [15]. Normal follicular growth necessitates a precise balance of multiple endocrine, paracrine, and autocrine components [15]. Any disturbance in this equilibrium can affect follicular growth, resulting in pathological conditions such as follicular cyst development [16].

Serum transudate and follicular fluid contain locally produced substances involved in follicular cells’ metabolic activities [8]. The latter and the properties of the blood-follicle barrier have been shown to differ.

Generally, follicular fluid is a yellow, semi-viscous complex fluid that promotes follicular cell differentiation and nuclear and cytoplasmic oocyte maturation. Its components are primarily derived from blood plasma, but it also contains factors produced locally by the granulosa and theca cells [17]. It contains oxygen, hormones, electrolytes, energy substrates, and metabolites [18]. In contrast, ovarian cystic fluid is dark and viscous [16]. It is characterized by increased levels of some elements that may indicate the degree of maturation of OC and reflect how they persist [19]. Glucose and metabolic hormones have been shown to regulate steroidogenesis directly at the ovarian level [20].

This study only reports significant differences in biochemical and hormonal parameters in glucose, insulin, cholesterol, and P4 concentrations between serum and follicular fluid (Table-1). Glucose concentrations in follicular and cystic fluids, on the other hand, were significantly lower than those in serum (Tables-1 and 2). This is consistent with the findings in camels [21], cattle [7], and buffaloes [22]. The cystic follicle microenvironment is caused by granulosa degeneration in cystic fluid with low glucose levels. Cows [14] and buffaloes [16] have yielded similar results. Similarly, a good correlation was found between serum and follicular fluid components, implying that hypoglycemia may reduce glucose levels in the follicles [7]. Glucose in follicular fluid is produced by glycolysis in granulosa cells and enters the follicle [7, 23]. Thus, low glucose levels in the cystic fluid are due to postmortem changes that can convert glucose to lactate by anaerobic glycolysis and a decrease in blood glucose flow. This is consistent with the findings from cyclic animal experiments [24]. Cysts in buffalo [16] and cattle [25] have lower glucose contents than pre-ovulatory follicles. This contradicts our findings of higher glucose serum levels (Tables-1 and 2) and what has been reported in she-camels with multiple OC [10].

Recent research reports that the mean glucose content in camel serum with different sized follicles in follicular and cystic fluids was significantly lower than its corresponding values. Female dromedaries previously demonstrated similar findings [26]. Our results show that serum cholesterol is significantly higher than follicular fluid cholesterol (Table-1). In females, cholesterol is the precursor of all steroid hormones, including estrogen and P4 [27, 28]. Cholesterol in follicular fluid is derived from granulosa cells and blood serum [29]. Cholesterol levels are also directly related to the animal’s energy state. The relationship between glucose metabolism and cholesterol levels in ruminants has been reported [27]. Cholesterol concentrations in follicular fluid were significantly lower than those in serum (Tables-1 and 2). Cattle showed similar trends [19, 30]. Serum cholesterol levels in camels were found to be 3.5 times higher than those in follicular fluid [21]. It has been proposed that blood cholesterol is not the most important metabolite for steroid synthesis and granulosa cells have many cholesterol esters that can provide cholesterol to ovarian function [21]. Furthermore, in dromedaries, cholesterol concentrations in blood serum were significantly higher in animals with extra-large follicles than in pre-ovulatory follicles [23]. Similar findings were observed in she-dromedary follicular fluids of pre-ovulatory and oversized follicles [23, 26].

Urea levels in follicular fluid and serum were not significantly different (Table-1). Similar results have been reported in cows [7, 14]. However, serum and cystic fluid urea levels differed (Tables-1 and 2). Urea concentrations were higher in fluid aspirated from developing follicles than in serum, most likely due to active transport or local urea synthesis by follicular cells [30, 31]. As a result, the elevated concentrations found in the cystic fluid may be due to abnormal OC production and/or excessive active transportation as a result of blood-follicle barrier dysfunction; this may explain why cystic cows’ sera have low urea levels in the presence of hypoglycemia seen in cystic animals. In this study, urea concentrations in follicular fluid were significantly lower than those in serum. This is in contrast to the reported results [14].

Serum total protein concentrations were significantly higher than follicular fluid (Table-1). Some authors discovered corresponding results [7, 30], and others estimated the average total protein concentration in the follicular fluid to be 75%–80% of serum [7, 14]. However, Wise [32] noted a high correlation between the amounts of total protein in these two liquids; he claims that total proteins in follicular fluid originate from blood serum, most likely through a mechanism similar to filtration. There was no statistically significant difference in protein content between follicular and cystic fluids. This contradicts the findings of Mimoune *et al*. [14] in cows. Because follicular fluid proteins are provided by blood and follicular secretions, changes in cystic fluid protein levels may cause changes in synthesis capacity, metabolism, and follicular wall construction, which may play a role in OC pathogenesis [17, 33]. The granulosa and follicular cells produce a large amount of P4. It acts as a precursor for androgen and, later, estrogen synthesis [34] and improves the synthesis of proteolytic enzymes required for follicle collapse during ovulation [35].

According to recent research, the mean concentration of P4 in follicular and cystic fluids does not vary significantly [14, 23]. The corpus luteum is the primary source of peripheral P4 in she-dromedaries. Therefore, in the absence of mating, P4 levels remain very low (<1 ng/mL) throughout the follicular wave [36, 37]. Because all she-dromedaries studied were not pregnant, low P4 levels in the blood serum (<1 ng/mL) were expected, although P4 concentrations in blood, follicular fluid, and cystic fluid were relatively high (Tables-1–3), indicating that the animals had mated shortly before slaughter. P4 concentrations in follicular fluid of oversized follicles of she-dromedaries were 330 times higher than in serum [23], and P4 concentrations in cystic follicles of buffaloes were higher than in normal pre-ovulatory follicles [16].

Local metabolism produces triglycerides. They are important sources of energy for oocyte maturation [29]. There was no statistically significant difference in triglyceride concentrations between follicular and cystic fluids. This has also been reported in cows [14] in Algeria and goats [29] in Kerala. However, in camels, signals of low triglyceride concentrations in oversized follicles have been expressed [38], resulting in a lower triglyceride level in sheep’s large follicles [31]. In this study, the triglyceride concentration level remained constant.

The insulin levels in the follicular fluid and blood serum differed significantly (Table-1), but there was no significant difference between the follicular and cystic fluids (Tables-1 and 2). This was also stated in the previous studies [19, 39, 40]. Insulin is involved in steroidogenesis by stimulating luteinizing hormone (LH) receptors in granulosa cells and indirectly stimulating the insulin-like growth factor-1 receptor during follicular development, maturation, and ovulation [19, 39]. Hypoinsulinemia may reduce androgen and estrogen production and alter the follicle’s ability to acquire LH receptors, inhibiting follicle growth and ovulation and encouraging follicle persistence as anovulatory structures [40]. Even after glucose administration, insulin production was reduced in cystic cows [41]. Recently, a decrease in insulin receptor (IR and IRS1) expression was observed [19, 40].

This study found no significant difference in creatinine concentrations and liver enzyme (AST, ALT, and GGT) activity between serum and follicular and cystic fluids (Tables-1 and 2). Camels [23] and cows [19] showed similar results.

The breed of the she-dromedaries studied had no effect on the levels of various biochemical and hormonal components in follicular and cystic fluids (Table-3). There were no significant changes in the main biochemical and hormonal contents of she-dromedary sera with different sized follicles (Table-3).

## Conclusion

One could conclude that OC develops and persists in a metabolic environment distinct from the follicular fluid. The findings highlight the need for additional research on the blood-follicle barrier and etiopathogenesis and conceptualization of cystic ovarian syndrome, to understand the regulatory mechanism that leads to the cyst formation.

## Authors’ Contributions

AB, MHB, and KM: Conceived and designed the study. AB, NM, BB, and MHB: Carried out the experiment and analyzed the data. KM and RK: Revised the manuscript. All authors have read and approved the final manuscript.
